# Bedside anti-Xa measurement for therapeutic assessment of a prophylactic anticoagulation regimen

**DOI:** 10.1186/cc12286

**Published:** 2013-03-19

**Authors:** B Dewulf, I Herck, F De Somer, K Francois, J Decruyenaere

**Affiliations:** 1 AZ Sint-Jan, Brugge, Belgium; 2 University Hospital, Gent, Belgium

## Introduction

Dose adjustments of low molecular weight heparin (LMWH) based on daily anti-Xa measurement by chromogenic assay remain controversial in daily clinical practice. One of the major obstacles is the cost of such a test. An affordable and reliable bedside test could change practice to an individual tailored dosing of LMWH. The aim of our study was to evaluate whether a prophylactic dose regimen of 40 mg enoxaparine in cardiac surgical patients increases the anti-Xa activity to the level necessary for efficient prevention of a thromboembolic event [1]. Secondarily we tested whether there was a reliable correlation between a bedside anti-Xa measurement compared with a two-stage chromogenic assay at the laboratory [2].

## Methods

This was an open, single-centre, prospective, nonrandomized clinical trial at a university hospital. All patients that needed prophylactic dosing of enoxaparine after cardiac surgery were duly informed and after giving written consent we included 44 patients with a mean Euroscore of 1.66. The demographic specifications, medical and surgical history of all patients were collected. Anti-Xa activity was measured at three different points in time. We determined baseline, peak and trough anti-Xa activity: preoperatively, and respectively 4 hours after the third dose of enoxaparine and 30 minutes before the fourth dose. Each measurement was done with both techniques, the two-stage chromogenic assay at the laboratory (Biophen^®^) and the bedside assay (Hemochron^® ^Jr).

## Results

Our dose regimen of enoxaparine achieved in one-half of the included patients a sufficient anti-Xa activity for prevention of thromboembolic events. One-half of the patients with insufficient anti-Xa activity had a body mass index over 30 kg/m^2^. Comparison of the bedside assay with the two-stage chromogenic assay by means of the Pearson's correlation coefficient showed correlation of the two tests if no variables were taken into account. In the Bland-Altman analysis we could not confirm this correlation.

## Conclusion

The bedside anti-Xa activity assay with a Hemochron device tends to show some correlation with the two-stage chromogenic assay, but insufficient to be used as an alternative, in this small but uniform patient population. Use of a standard dosing protocol for enoxaparine administration is prone for underdosage in post-cardiac surgery patients and may increase postoperative morbidity.
